# Fast Sample Adaptive Offset Jointly Based on HOG Features and Depth Information for VVC in Visual Sensor Networks

**DOI:** 10.3390/s20236754

**Published:** 2020-11-26

**Authors:** Ruyan Wang, Liuwei Tang, Tong Tang

**Affiliations:** 1 School of Communication and Information Engineering, Chongqing University of Posts and Telecommunications, Chongqing 400065, China; wangry@cqupt.edu.cn (R.W.); s180101091@stu.cqupt.edu.cn (L.T.); 2 Key Laboratory of Optical Communication and Networks, Chongqing 400065, China; 3 Key Laboratory of Ubiquitous Sensing and Networking, Chongqing 400065, China

**Keywords:** visual sensor networks, versatile video coding, sample adaptive offset, edge offset, depth

## Abstract

Visual sensor networks (VSNs) can be widely used in multimedia, security monitoring, network camera, industrial detection, and other fields. However, with the development of new communication technology and the increase of the number of camera nodes in VSN, transmitting and compressing the huge amounts of video and image data generated by video and image sensors has become a major challenge. The next-generation video coding standard—versatile video coding (VVC), can effectively compress the visual data, but the higher compression rate is at the cost of heavy computational complexity. Therefore, it is vital to reduce the coding complexity for the VVC encoder to be used in VSNs. In this paper, we propose a sample adaptive offset (SAO) acceleration method by jointly considering the histogram of oriented gradient (HOG) features and the depth information for VVC, which reduces the computational complexity in VSNs. Specifically, first, the offset mode selection (select band offset (BO) mode or edge offset (EO) mode) is simplified by utilizing the partition depth of coding tree unit (CTU). Then, for EO mode, the directional pattern selection is simplified by using HOG features and support vector machine (SVM). Finally, experimental results show that the proposed method averagely saves 67.79% of SAO encoding time only with 0.52% BD-rate degradation compared to the state-of-the-art method in VVC reference software (VTM 5.0) for VSNs.

## 1. Introduction

Recently, the advances in imaging and micro-electronic technologies enable the development of visual sensor networks (VSNs) [1,2]. By integration of low-power and low-cost visual sensors, VSNs can obtain multimedia data such as images and video sequences. As the key applications in VSNs, video transmission and compression technology have been increasingly used in the field of communication and broadcasting. Especially with the development of Internet of Things [3,4,5,6] and 5G techniques [7,8], the transmission of video and multimedia information in mobile communication have become the current hot technology, and improving the compression performance of mobile videos could combine the mobile application with communication better in VSNs. Due to the increasing pressure of video storage and transmission [9,10], more and more efficient video coding standards have been put out in the last few decades. High-Efficiency Video Coding (HEVC/H.265) [11] is developed by Joint Collaborative Team of Video Coding (JCT-VC). Compared with advanced video coding (AVC/H.264), HEVC achieves equivalent subjective video quality with approximately 50% bit rate reduction. As the upcoming standard with the most advanced video coding technology, versatile video coding (VVC/H.266) [12,13] can reduce the bit rate by 40% while maintaining the same quality compared to HEVC. Therefore, it is very suitable for high-resolution and different formats of videos in VVC, such as virtual reality (VR) video [14] and ultra high-definition video [15]. However, block-based coding structures and quantization structures are still inherited, which cause artifacts in VVC, such as blocking artifacts, ringing artifacts, and blurring artifacts [16]. In order to reduce the ringing artifacts and distortions, VVC also adopts the sample adaptive offset (SAO) filter as in HEVC [17,18]. The thought of SAO is to reduce the distortion between the original samples and reconstructed samples by conditionally adding an offset value to each sample inside coding tree unit (CTU) [18]. Although the SAO process effectively improves the coding quality, it brings computational redundancy [19] as SAO not only refers to each original sample and reconstructed sample to collect statistic data, but also uses recursive rate distortion optimization (RDO) calculation to select the best SAO parameters [20].

Moreover, the coding complexity of VVC has increased greatly at the same time, which may be four to five times more complex than the current HEVC video coding standard [21,22,23]. Moreover, new video applications in VSNs need more bandwidth and less delay [24] when transmitting wireless communication, which brings great challenges to video coding and transmission in VSNs. Therefore, reducing the coding complexity of VVC becomes an important issue for VSNs. Thus, this paper proposes a SAO acceleration method to reduce the SAO coding time, thereby reducing the coding complexity of VVC and improving the efficiency of video transmission for VSNs. The main contributions of this paper can be summarized as follows.

(1) A new depth-based offset mode selection scheme of SAO is proposed for VVC. According to the partition depth of CTU, the edge offset (EO) mode and the band offset (BO) mode are adaptively selected.
(2) A histogram of an oriented gradient (HOG) feature-based directional pattern selection scheme is proposed for EO mode. The HOG features [25] of CTU are extracted and input to the support vector machine (SVM). The best directional pattern is output, skipping the RDO calculation process and sample collection statistics of the other three directional patterns.

The rest of the paper is organized as follows. Section 2 introduces the related work. Section 3 describes the overview of SAO algorithm in VVC. Section 4 introduces the proposed method. Experimental results are shown in Section 5. Section 6 concludes this paper.

## 2. Related Work

In recent years, many researchers have proposed improved methods to reduce the computational complexity of SAO. They can be classified into two categories: The first category focuses on reducing the complexity by improving the SAO algorithm directly. Joo et al. [26] proposed a fast parameter estimation algorithm for SAO by using the intra-prediction mode information in the spatial domain instead of searching all EO patterns exhaustively to simplify the decision of the best edge offset (EO) pattern. Furthermore, they also proposed to make a simplified decision of the best SAO edge offset pattern by using the dominant edge direction [27], which reduced the RDO calculation and sped up the SAO encoding process in HEVC. Zhang et al. [28] proposed to distinguish videos according to texture complexity and performed an adaptive offset process by reducing some unnecessary sample offsets to improve the video coding efficiency. Gendy et al. [29] proposed an algorithm to reduce the complexity of SAO parameter estimation by adaptively reusing the dominant mode of corresponding set of CTUs, which saved SAO encoding time. Although the above two methods save SAO encoding time, the BD-rate gain loss is large. Sungjei Kim et al. [30] proposed to decide the best SAO parameters earlier by exploiting a spatial correlation between current and neighbor SAO types, which reduced the parameter calculation of other SAO patterns.

The other category focuses on reducing the complexity of SAO by parallel processing on a central processing unit (CPU) and graphic processing unit (GPU). Zhang et al. [31] designed the corresponding parallel algorithms for SAO by exploiting GPU multi-core computing ability, and a parallel algorithm of statistical information collection, calculation of the best offset and minimum distortion, and SAO merging was proposed. D. F. de Souza et al. [32] optimized the deblocking filter and SAO by using GPU parallelization in a HEVC decoder for an embedded system. Wang et al. [33] redesigned the statistical information collection part, which computes offset types and values, to make it well suitable for GPU parallel computing. Later, Wang et al. [34] designed the pipeline structure of HEVC coding through the cooperation of CPU and GPU. Through the joint optimization of deblocking filter and SAO, the parallelism can be improved and the computational burden of CPU can be reduced.

Although the above methods have achieved good results in the research of SAO acceleration, these technologies are all designed for HEVC, and many new technologies have been added to VVC, such as multi-tree partitioning, independent coding of luma and chroma component, Cross-Component Linear Prediction (CCLM) prediction mode, Ref. [35,36,37] Position Dependent intra Prediction Combination (PDPC) technology, and so on [38,39]. These new technologies cause different encoding characteristics between VVC and HEVC. Therefore, the acceleration method of SAO for VVC needs to be re-studied.

**Figure 1:**
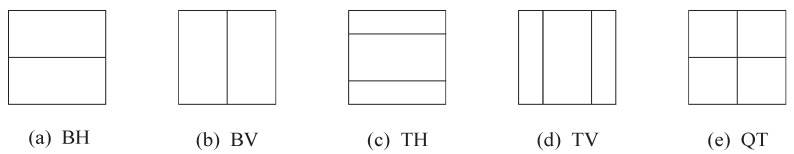
Five partition structures of quad-tree with nested multi-type tree (QTMT).

**Figure 2:**
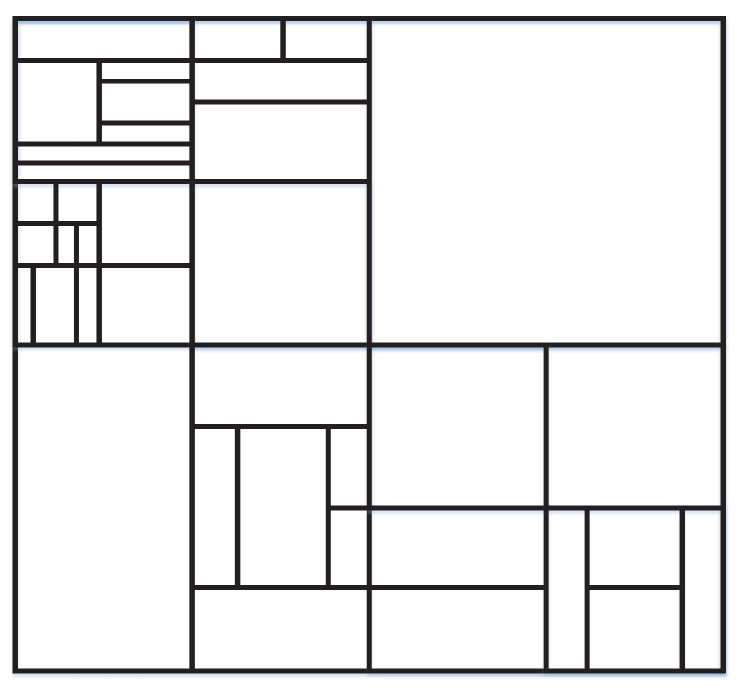
An example of QTMT structure in versatile video coding (VVC).

It should be noted that the proposed method utilizes depth information for SAO acceleration. Although the work in [26] is also depth-based, it is designed for HEVC. The block partition mode of VVC has large differences compared with that of HEVC. Concretely, VVC adopts the new quad-tree with nested multi-type tree (QTMT) [35,36]. Similar to HEVC, each frame is first divided into CTUs, and then further divided into smaller coding units (CUs) of different sizes. In the QTMT structure, there are five ways to split blocks, including horizontal binary tree (BH), vertical binary tree (BV), horizontal ternary tree (TH), vertical ternary tree (TV), and quad-tree (QT), and the five possible partition structures are shown in Figure 1. This division pattern means that the shape of CU includes square and rectangular. In VVC, the maximum value of CU partition is 128 × 128, and the minimum depth value is 0; the minimum partition of CU is 4 × 4, and the maximum depth is 6. In Figure 2, a possible CTU partitioning with the QTMT splits is depicted.

## 3. Overview of SAO in VVC

SAO, as a key technology of loop postprocessing, mainly consists of three steps: sample collection statistics, mode decision and SAO filtering. First, in the process of sample statistics collection, eight SAO offset patterns of each CTU need to be traversed, including four EO patterns, one BO pattern, two merge patterns, and one SAO off pattern. We need to traverse all possible offset patterns to collect statistic information. Then, for the mode decision, the encoder will perform RDO calculation for each pattern by the statistical data. We choose the best pattern according to the RDO calculation results of the eight patterns. Finally, in the filtering process, we add an offset value for each reconstructed sample.

SAO consists of two types of algorithms: Edge Offset (EO) and Band Offset (BO). The two methods are described as follows.

### 3.1. Edge Offset (EO)

**Figure 3:**
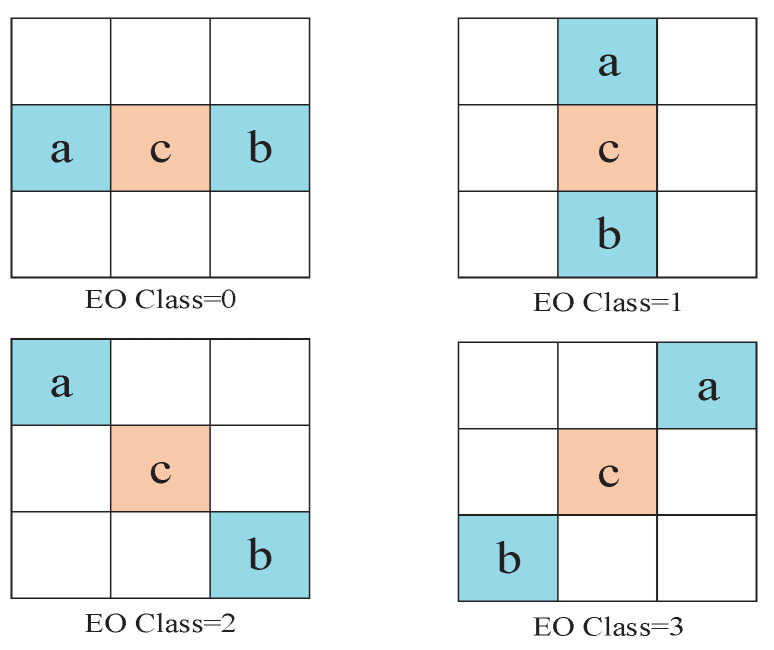
Four 1-D 3-pixel patterns for Edge Offset (EO) sample classification: horizontal (EO Class = 0), vertical (EO Class = 1), $135^{\circ }$ diagonal (EO Class = 2), and $45^{\circ }$ diagonal (EO Class = 3).

EO classifies samples based on direction, using four one-dimensional directional patterns: horizontal (EO$\_0^{\circ }$), vertical (EO$\_90^{\circ }$), 135 diagonal (EO$\_135^{\circ }$), and 45 diagonal (EO$\_45^{\circ }$). As shown in Figure 3, “c” represents the current sample, and “a” and “b” are two adjacent samples. The classification of the current sample “c” is based on the comparison between “c” and the two neighboring samples of it.

For a given EO pattern, samples are divided into five categories according to the relationship between the current pixel and neighbor pixels. Table 1 summarizes the classification rules for each sample. The offset values are always positive for categories 1 and 2, and negative for categories 3 and 4, which indicates that EO tries to reduce the distance between current sample and neighbor ones.

### 3.2. Band Offset (BO)

**Figure 4:**
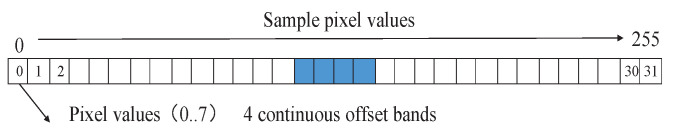
Illustration of band offset mode.

BO divides pixel range into 32 bands where each band contains pixels in the same intensity interval. Each interval band’s offset value is an average difference between original and reconstructed samples. Moreover, four consecutive bands are selected to calculate the differences in pixel values between original samples and reconstructed samples. Only four offsets of the consecutive bands are selected and signaled to the decoder. The schematic diagram of BO mode is shown in Figure 4.

## 4. Proposed SAO Method

In this section, first the motivation of the proposed method is analyzed. Next, the simplification scheme of offset mode selection is introduced. Then, the simplification scheme of EO mode is presented. Finally, the process of the proposed method is summarized with a flowchart.

### 4.1. Motivation

For the BO mode, the pixel values of the compensated samples are concentrated in the four consecutive bands, and the BO mode performs better for those regions where the pixel values are concentrated in small ranges. Therefore, the BO mode can be fast selected according to the pixel distribution of different CTUs.

For the EO mode, when it compensates for high-frequency distortion caused by quantization, it references neighbor pixels for pattern selection. Therefore, the best EO pattern is closely related to the main local edge features. In local areas, consecutive samples along the local edge direction are more probable to have similar values compared to other samples. The HOG feature is a feature descriptor used for object detection in computer vision and image processing. It is formed by calculating and counting the gradient direction histogram of the local area of the images. Therefore, the HOG features extracted from CTUs can be used to select the optimal pattern. The extracted HOG features are used as the input of the SVM, and the best EO pattern is directly selected by the output of SVM.

### 4.2. Simplification of Offset Mode Selection

**Figure 5:**
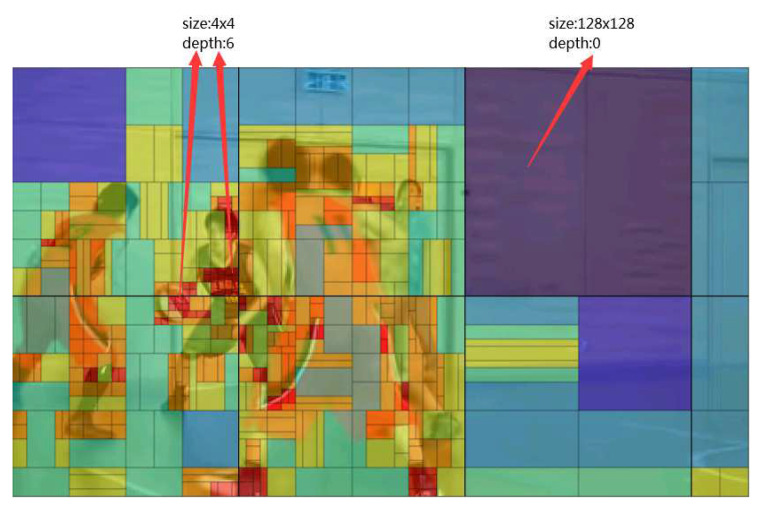
Coding unit (CU) partition of the 87th of BasketballPass encoded with QP = 22 under all intra (AI) configuration.

**Figure 6:**
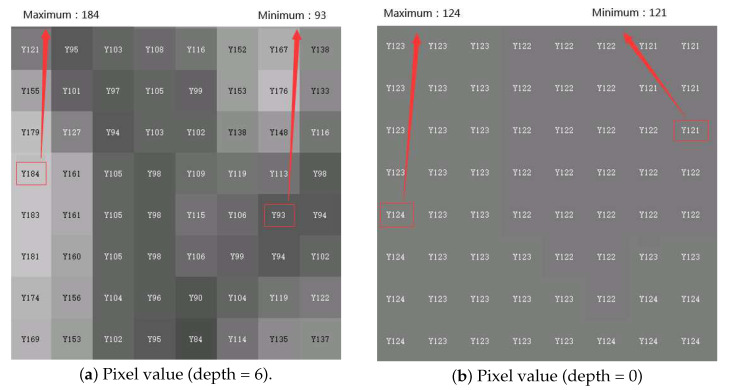
Pixel value distribution comparison between complex region and simple region.

The BO mode works in the areas where the pixel values are concentrated, and the pixel distribution is closely related to picture contents. We observe that regions with complex texture have complex pixel distribution and the corresponding pixel values are decentralized. On the contrary, regions with simple texture have concentrated pixel distribution. Simultaneously, regions with complex texture are usually encoded with small CU, and regions with simple texture are usually encoded with large CU. For example, Figure 5 depicts the partition result of each CTU in the 87th frame of the sequence BasketballPass under all intra (AI) configuration, where the quantization parameter (QP) is 22. We can see that larger CU is selected for encoding flat areas, such as floors and walls. A smaller CU is selected to encode regions with complex texture, such as the human head and a basketball. Moreover, to show the pixel distribution difference between complex region and simple region, a block belongs to complex region is selected and the pixel distribution is shown in Figure 6a, and a block belongs to simple regions is selected and the pixel distribution is shown in Figure 6b. It can be seen that for block belongs to complex regions (Figure 6a), the pixel values are decentralized (minimal pixel 93, maximum pixel 184). For block belongs to simple regions(Figure 6b), the pixel values are concentrated (minimal pixel 121, maximum pixel 124).

Therefore, the partition depth of CTU can be used to measure the pixel distribution, where the depth of CTU represents the maximum depth of CU in CTU. Concretely, smaller depth means simpler texture, which indicates more concentrated pixel distribution. Therefore, CTU with small depth can directly choose BO mode as the offset mode, because all the concentrated pixels of this CTU could be covered by four consecutive bands. On the other hand, CTU with large depth contains decentralized pixel range. If BO mode is adopted, many samples can not be covered by the four consecutive bands, which will degrade offset performance. Therefore, in this paper, if depth < δ, the BO mode is selected as the SAO type; otherwise, the EO mode is selected.

**Figure 7:**
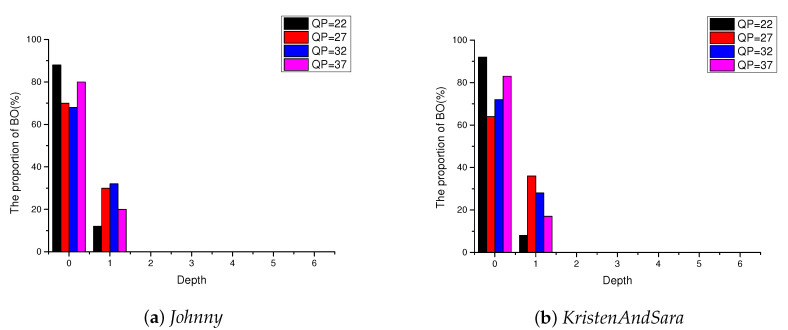
Proportion of each depth in which the best mode is BO mode between EO mode and BO mode under LB (The test sequences are *Johnny* and *KristenAndSara*, and the test frames are 100 for each sequence).

Obviously, threshold δ directly influences the performance of the proposed method. Thus, we conduct some experimental tests to select a proper value for δ. We count the proportion of each depth in which the best mode is BO mode between EO mode and BO mode, where the test sequences are Johnny and KristenAndSara under low delay with B picture (LB) configuration, and the number of test frames is 100 for each sequence. As shown in Figure 7, the depth values are mostly concentrated at 0 and 1 when the best mode is BO mode, which illustrates that BO mode shows good effect in the area with lower complexity, and EO mode performs better in the area with higher complexity. Therefore, we set δ=2. If the depth <2, BO mode will be directly selected as the best mode between EO mode and BO mode. Otherwise, EO mode will be directly selected as the best mode.

### 4.3. Simplification of EO Mode

This section describes the simplification of EO mode. First, the gradient computation is introduced. Then, the HOG features calculation is present. Finally, the classification based on SVM is analyzed.

#### 4.3.1. Gradient Computation

The HOG features are extracted by calculating and counting the histogram of the gradient direction of the local area of the picture. Therefore, we divide the CTU picture into small cells to calculate the gradient amplitude and gradient direction. The Scharr operator and Sobel operator are two common operators for computing gradient. The principles and structures of the two operators are similar. The central element of the Scharr operator takes more weight, so the accuracy calculated by it is higher. Therefore, in this paper, we choose the Scharr operator to calculate the edge gradient. The direction gradient of Scharr operator is calculated as

(1) $$\begin{array}{c} \begin{array}{cc} Gx_{i,j} & =3f_{i+1,j-1}+10f_{i+1,j}+3f_{i+1,j+1}\\ \; & -3f_{i-1,j-1}-10f_{i-1,j}-3f_{i-1,j+1} \end{array} \end{array}$$

(2) $$\begin{array}{c} \begin{array}{cc} Gy_{i,j} & =3f_{i-1,j-1}+10f_{i,j-1}+3f_{i+1,j-1}\\ \; & -3f_{i-1,j+1}-10f_{i,j+1}-3f_{i+1,j+1} \end{array} \end{array}$$

where $Gx_{i,j}$ and $Gy_{i,j}$ represent the gradient in the horizontal direction and the gradient in the vertical direction, respectively, and the gradient amplitude can be roughly estimated in the following ways,

(3) $$\begin{array}{c} Amp\left(\left\{Gx_{i,j},Gy_{i,j}\right\}\right)=\sqrt{{\left(Gx_{i,j}\right)}^{2}+{\left(Gy_{i,j}\right)}^{2}} \end{array}$$

The decision of EO pattern is made by horizontal gradient and vertical gradient, and the HOG is established by $\frac{Gy_{i,j}}{Gx_{i,j}}$. Let $\eta =\frac{Gy_{i,j}}{Gx_{i,j}}$, and the direction angle of the gradient $\theta_{i,j}$ could be calculated as follows.

(4) $$\theta_{i,j}=arctan\left(\eta \right)$$

#### 4.3.2. HOG Features Calculation

**Figure 8:**
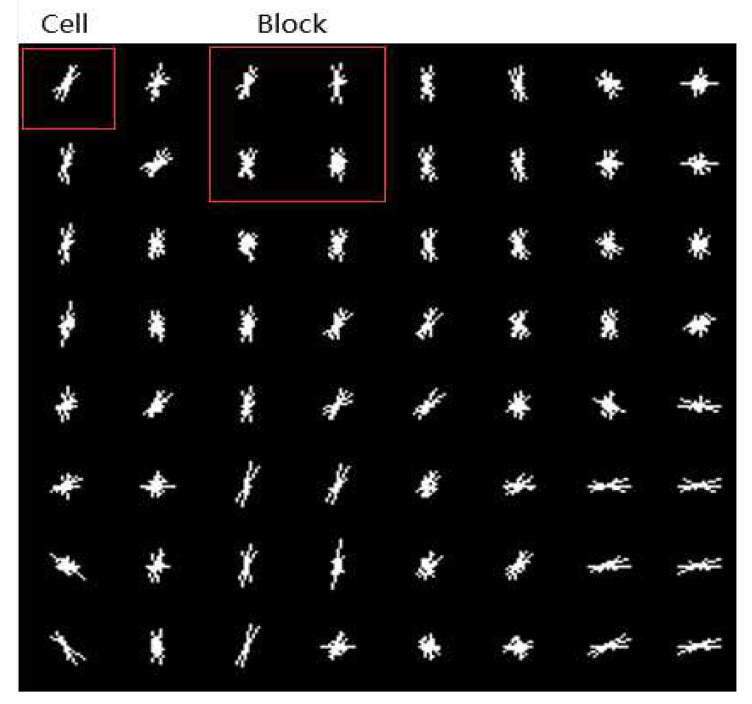
Visualization of histogram of an oriented gradient (HOG) features.

In the process of HOG features calculation, each pixel in the cell votes for a direction-based histogram channel. According to the gradient direction and gradient amplitude of each pixel in the cell, the gradient amplitude value is added to the histogram channel to which the current pixel belongs. The histogram channels are evenly distributed in the range of 0–$180^{\circ }$ or 0–$360^{\circ }$. EO modes are classified according to four kinds of position information (horizontal, vertical, $135^{\circ }$ diagonal, and $45^{\circ }$ diagonal) between current pixel and neighbor pixels, and we divide 0–$180^{\circ }$ into 9 bins ($20^{\circ }$ for each part) as histogram channels. Due to changes in local illumination, the range of gradient intensity is very large. Therefore, groups of adjacent cells are considered as spatial regions called blocks to perform normalization operations to achieve better extraction results, and the histograms of many cells in the block represent the block histograms, which represent the feature descriptor. After the calculation of block gradient histograms, all the block gradient histograms in a CTU represent all the features within the CTU, and all the block feature vectors are concatenated to form the final feature vectors in each CTU. Figure 8 shows the visualization of a picture based on HOG features, where 4 cells form a block.

#### 4.3.3. Classification Based on SVM

The best pattern is predicted by SVM. The SVM algorithm is to find the best hyperplane in a multidimensional space as a decision function, so as to achieve classification between classes. For a given training set, $S=\{\left(x_{i},y_{i}\right)\}$, $x_{i}$ represents the feature vector of the training samples, $y_{i}$ represents the label of the training samples, and $y_{i}=1$ and $y_{i}=-1$ denote the positive and negative samples respectively. Therefore, hyperplane f(x) can be calculated as follows,

(5) $$\begin{array}{c} \begin{array}{cc} f(x) & =\omega^{T}x+b\\ \; & =\sum_{i=1}^{m}\alpha_{i}y_{i}x_{i}^{T}x+b \end{array} \end{array}$$

where ω is the normal of the hyperplane, *m* is the number of support vectors, $\alpha_{i}$ is the Lagrange multiplier and *b* is the deviation. The objective function can be calculated as follows,

(6) $$\min_{\omega ,b,\xi_{i}}\;\;\;\;\;\frac{1}{2}{∥\omega ∥}^{2}+C\sum_{i=1}^{m}\xi_{i}\;$$

(7) $$\begin{array}{c} s.t.\;\;\;\;\;y_{i}\left(\omega^{T}x_{i}+b\right)\geq 1-\xi_{i}\\ \;\;\;\;\;\;\;\;\;\;\;\;\;\;\;\;\;\xi_{i}\geq 0,\;\;\;\;\;i=1,2,\ldots ,m. \end{array}$$

where the $\xi_{i}$ is the slack variable, and C is the penalty factor. The kernel function $\kappa (x,x_{i})$ is used to map the original space to a higher dimensional space, and f(x) can be rewritten as

(8) $$f\left(x\right)=\sum_{i=1}^{m}\alpha_{i}y_{i}\kappa \left(x,x_{i}\right)+b$$

In this paper, we choose radial basis function as the kernel function. The kernel function can be calculated as

(9) $$\kappa \left(x,x_{i}\right)=\exp\left(-\gamma {∥x-x_{i}∥}^{2}\right),\;\;\;\gamma >0$$

where γ defines the impact of a single sample.

The samples can be classified according to the obtained hyperplane. In this paper, there are four directional patterns of EO as candidates for SAO. Therefore, we design four one-versus-rest SVM models. For each model, the positive examples are the CTUs with the best EO pattern, and the negative examples are the CTUs with the remaining three other patterns. The HOG features of CTUs are used as the input of SVM to train the four SVM models off-line, and the best EO pattern is directly selected through the models.

### 4.4. Summary

**Figure 9:**
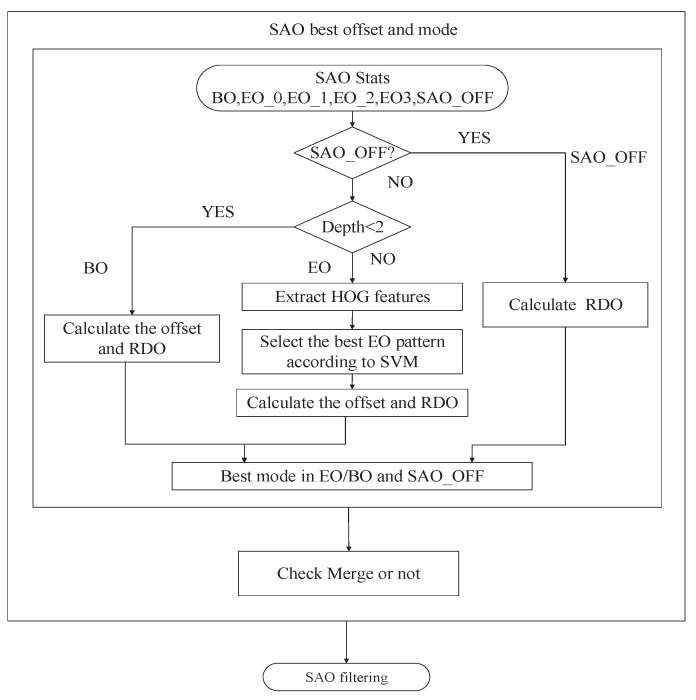
Flowchart of proposed overall sample adaptive offset (SAO) algorithm.

Combining the simplification of offset mode selection and directional pattern selection, the flowchart of the proposed SAO acceleration method is summarized in Figure 9. Concretely, first, the depth information is used to evaluate the pixel distribution, and SAO is accelerated based on the depth information. If the depth is smaller than 2, BO mode is selected as the best offset mode; otherwise, EO mode is selected as the best offset mode. Second, for the EO mode, the HOG features of each CTU are extracted, and the best directional pattern of EO mode is predicted based on HOG features and SVM. Next, the best mode is selected by comparing the RDO values of EO or BO mode and SAO off state. Then, we compare this mode with the best pattern in SAO merge mode to select the final offset mode and obtain the SAO offset information. Finally, the offset value is added to the reconstructed samples.

## 5. Experimental Result

In this section, first the experimental design is introduced. Then, the performance of HOG and SVM is analyzed. Finally, the acceleration performance of different methods is compared.

### 5.1. Experimental Design

The proposed method and other acceleration methods are implemented on the VVC reference software (VTM5.0) [40]. The test platform is a Dell R730 server, which has two 12-core Intel(R) Xeon(R) E5-2620 V3 CPUs with a main frequency of 2.4 GHz made in China. Our experimental materials are from the standard sequences of JCT-VC proposals [41], as shown in Table 2. All the experimental sequences are encoded with four modes, which contains AI mode, random access (RA) mode, low delay with P picture (LP) mode, and LB mode. The main encoding parameter configurations are listed in Table 3.

### 5.2. Effectiveness Verification of HOG Features

The parameters and data regarding the HOG features are calculated and shown. The first sequence in each class is selected as the training sequences, and the remaining sequences are used as the testing sequences. In this paper, a self-made data set is used. For training of each configuration, we select a total of about 16,000 CTUs of each training sequence for each QP, and we extract the HOG features of CTUs in the training sequences to make the data set. The size of the CTU is 128 × 128 (For the CTUs whose size is not 128 × 128 at the boundary, pixels of boundary are used to fill the size to 128 × 128 when calculating the HOG features). The cell size of the extracted HOG features extraction is 8 × 8, and the size of each block is 16 × 16. The size of blockStride is 16 × 16, so there is no overlap among the blocks. We divide 0–$180^{\circ }$ into 9 parts ($20^{\circ }$ for each part) as histogram channels. The radial basis function (RBF) is selected as the kernel function of SVM, and the penalty factor C is set to 10 and the parameter γ is set to 0.09.

**Figure 10:**
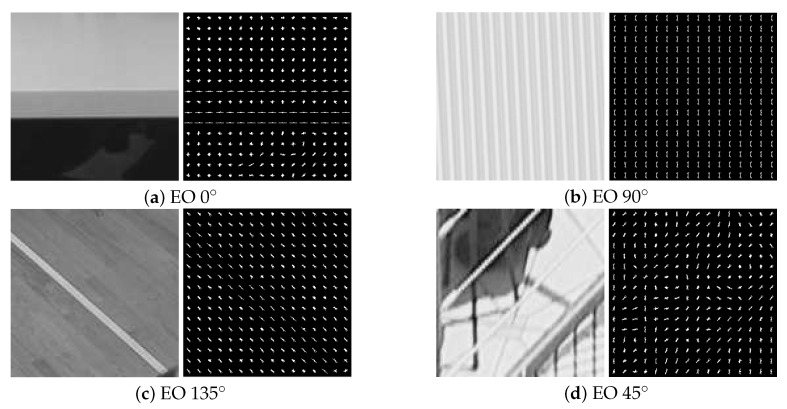
Coding tree units (CTUs) and HOG features maps of each EO pattern.

Figure 10 shows the CTU pictures for each best EO pattern and their corresponding HOG features maps. The four EO patterns correspond to the four labels of SVM, and the HOG features of each CTU are used as the input of SVM to predict.

**Figure 11:**
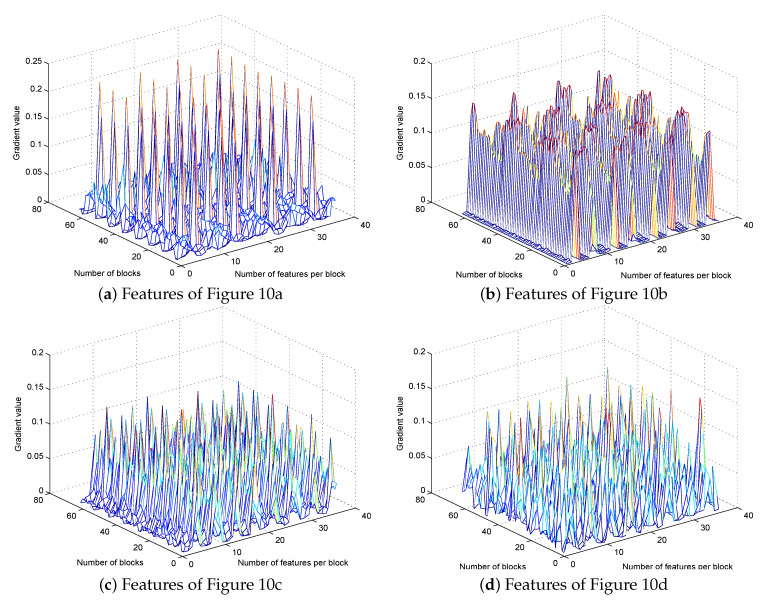
HOG features and the gradient value of each feature of Figure 10a–d.

In this paper, each CTU contains 8 × 8 (64) blocks, while each block contains 4 cells, and each cell is divided into 9 bins. Therefore, a CTU contains a total of 8 × 8 × 4 × 9 (2304) dimensional features. Figure 11a–d shows the corresponding features and the gradient value of each feature of Figure 10a–d.

Taking these features as the input of SVM, the best EO pattern can be predicted directly by comparing the probability that the current CTU belongs to each EO pattern. Table 4 shows the prediction accuracy of EO Pattern in this paper and the works in [26,27]. Compared with the methods in [26,27], the algorithm based on HOG features fully combines the features of images and shows higher prediction accuracy.

### 5.3. Acceleration Performance Comparison

**Figure 12:**
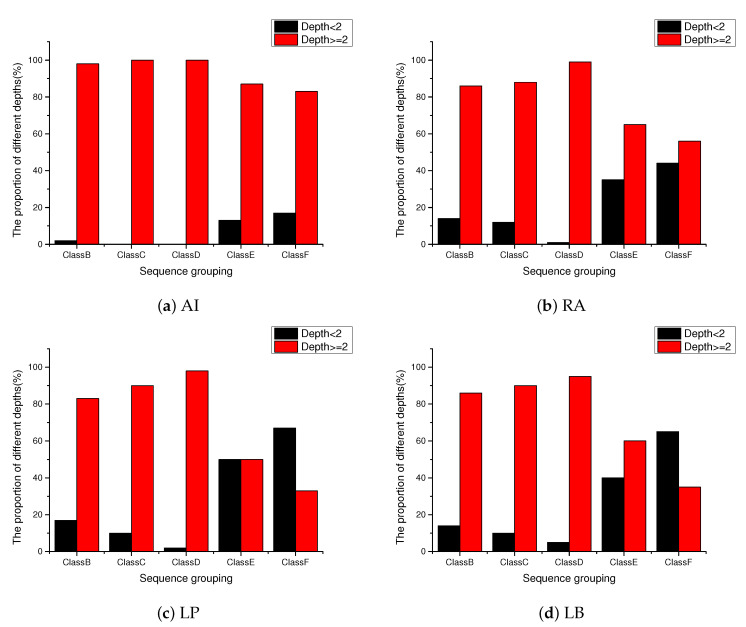
The proportion of depths of all testing sequences encoded with four configurations.

Figure 12 summarizes the distribution of depth values of CTUs under different configurations. From the figure, it can be seen that for the sequences with higher texture complexity, the depth of CTU is larger, such as the sequences in ClassB, ClassC, and ClassD; for those sequences with lower texture complexity, there are more CTUs with smaller depth than the sequences with high texture complexity, such as the sequences in ClassE and ClassF. This is also in line with our expectations.

We evaluate the performance of the algorithm with the Bjϕntegaard [42] metric (BD-rate) and the reduction of SAO encoding time ΔT. ΔT can be calculated as follows.

(10) $$\Delta T=\frac{T_{VVC(SAO)}-T_{Proposed(SAO)}}{T_{VVC(SAO)}}$$

Table 5 summarizes the BD-rate and run time reduction of the proposed method compared with VTM5.0. The result shows that the proposed method averagely achieves 63.68%, 65.09%, 71.46%, and 70.93% SAO encoding time saving with 0.20%, 0.33%, 0.96%, and 0.59% coding performance gain degradation for AI, RA, LP, and LB, respectively. The comparison shows that the proposed method can effectively reduce the SAO encoding time in the case of a small BD-rate performance loss for VSNs.

Table 6 compares the proposed method with the other three methods in [27,28,30] under AI configuration. It can be seen that the proposed method averagely achieves 63.68% SAO encoding time saving, which is better than the methods in [27] (53.43%), [28] (9.62%), and [30] (48.34%). Compared with the method in [27], it can be seen that the proposed algorithm achieves further computational complexity reduction with a much smaller increment in the BD-rate. This is because that the prediction accuracy of the best EO pattern of the method by HOG features is more accurate than that of the [27] directly using Sobel operator. In addition, this paper further optimizes the coding efficiency by combining the depth. Compared with the method in [28], we can see that the SAO encoding time saved in this paper in VVC is much more than that in [28]. On the one hand, the algorithm in [28] directly uses the depth information to turn on SAO adaptively. Due to the block partition method based on QTMT, there are more choices of block partition in VVC, which leads to great differences in block partition structure between VVC and HEVC. On the other hand, the optimization of EO mode is not considered in [28]. Compared with the method in [30], it can be seen that the SAO encoding time saved in this paper in VVC is more than that in [30] with almost the same BD performance loss. This is because in [30] at least two patterns of EO mode and BO mode must be calculated. Moreover, when the best mode is SAO off, all possible SAO patterns must be calculated, which consumes a lot of time.

**Figure 13:**
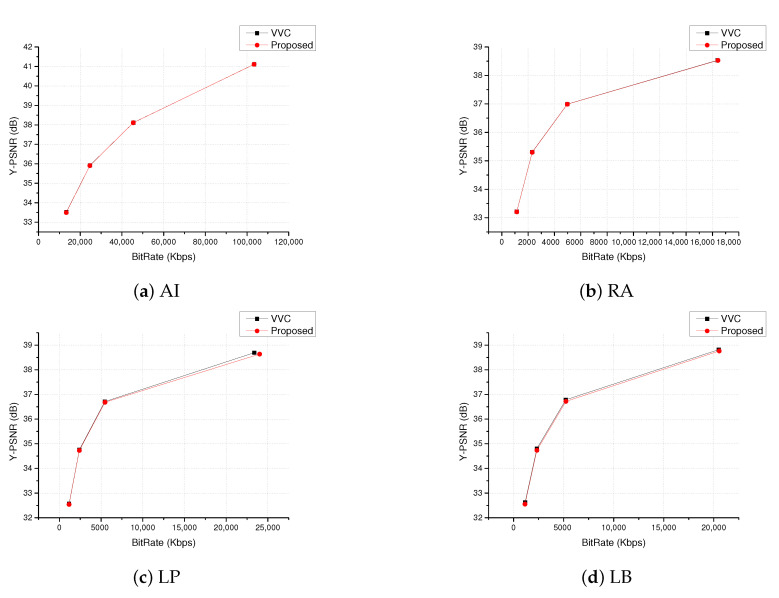
RD curve comparison of Cactus.

Figure 13 evaluates the rate–distortion curves of Bit Rate and PSNR of Cactus sequence in AI, RA, LP, and LB. It can be seen that the two curves almost coincide, which indicates that the encoding performance of the fast SAO algorithm proposed in this paper is similar to the default algorithm of VVC in terms of objective quality. It means that the proposed algorithm greatly reduces the SAO encoding time and improves the encoding efficiency with almost no lose of SAO encoding quality for VSNs.

**Figure 14:**
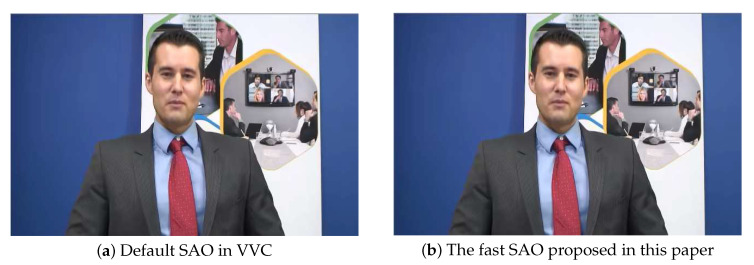
Comparison of the decoding picture of the 45th of Johnny.

**Figure 15:**
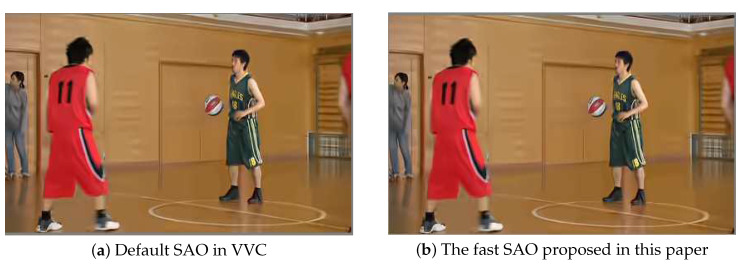
Comparison of the decoding picture of of the 3rd of BasketballPass.

Figure 14 and Figure 15 compare the subjective quality from Johnny and BasketballPass by using the default algorithm in VVC and our algorithm in this paper, and we analyze the situation when QP is 22 under AI configuration. As shown in Figure 14 and Figure 15, the differences of subjective quality between the two algorithms are also barely visible to the naked eye, which shows that the subjective quality loss caused by the algorithm in this paper can be ignored.

## 6. Conclusions

Complex calculation of SAO is a bottleneck to realize real-time transmission for VVC in VSNs. In order to solve the time-consuming problem of the SAO encoding process in VVC, this paper proposes a fast sample adaptive offset algorithm jointly based on HOG features and depth information for VSNs. First, the depth of each CTU is utilized to simplify the offset mode selection. Then, for EO mode, the HOG features and SVM are used to predict the best pattern in EO mode, skipping 75% calculation of the mode selection in EO mode. Finally, experimental results show that the proposed method can reduce the SAO encoding time by 67.79% with negligible objective and subjective degradation compared with the state-of-the-art method in VVC reference software, which is meaningful for real-time encoding applications in VSNs. In addition, in our future work, finding the optimization method of BO in VVC and making it suitable with all patterns and sequences will be studied.

## Figures and Tables

**Table 1:** Sample classification rules for EO.

Category	Conditions
1	c<a&&c<b
2	(c<a&&c==b)‖(c==a&&c<b)
3	(c>a&&c==b)‖(c==a&&c>b)
4	c>a&&c>b
0	None of above

**Table 2:** Experimental test materials.

Class	Resolution	Sequence Name	Encoded Frames	Frame Rate
ClassB	1920 × 1080	BQTerrace	120	60 fps
	1920 × 1080	Cactus	100	50 fps
	1920 × 1080	Kimono1	100	24 fps
ClassC	832 × 480	BasketballDrill	100	50 fps
	832 × 480	PartyScene	100	50 fps
	832 × 480	BQMall	120	60 fps
ClassD	416 × 240	BQSquare	120	60 fps
	416 × 240	BasketballPass	100	50 fps
	416 × 240	BlowingBubbles	100	50 fps
ClassE	1280 × 720	Fourpeople	120	60 fps
	1280 × 720	Johnny	120	60 fps
	1280 × 720	KristenAndSara	120	60 fps
ClassF	1280 × 720	SlideEditing	100	30 fps
	1280 × 720	SlideShow	100	30 fps

**Table 3:** Main encoding parameter configurations.

Codec	VTM5.0
Configurations	All Intra (AI) Random Access (RA) Low delay with P picture (LP) Low delay with B picture (LB)
Profile	Main
GOPsize	8
Quantization Parameter	22, 27, 32, and 37
Deblock Filter	ON
Adaptive Loop Filter	OFF

**Table 4:** Prediction accuracy comparison of different SAO methods.

Algorithm	Accuracy(%)
Intra-based EO [26]	72.20
Sobel-based EO [27]	76.30
Proposed	79.06

**Table 5:** BD performance and time reduction of the proposed method encoded with AI, RA, LP, and LB configurations.

Anchor(VTM5.0) SAO Disabled with 128 × 128 CTU		Y BD-Rate	ΔT
All Intra (AI)	ClassB	0.15%	63.22%
	ClassC	0.03%	62.87%
	ClassD	−0.04%	63.01%
	ClassE	0.10%	64.43%
	ClassF	0.77%	64.87%
Random Access (RA)	ClassB	0.44%	61.68%
	ClassC	0.11%	64.44%
	ClassD	0.09%	63.87%
	ClassE	0.22%	67.20%
	ClassF	0.79%	68.28%
Low Delay P (LP)	ClassB	1.17%	69.88%
	ClassC	0.71%	69.10%
	ClassD	0.36%	68.72%
	ClassE	1.49%	73.59%
	ClassF	1.05%	76.01%
Low DelayB (LB)	ClassB	0.77%	69.54%
	ClassC	0.48%	68.99%
	ClassD	0.08%	68.55%
	ClassE	0.52%	72.48%
	ClassF	1.08%	75.10%
Summary	AI	0.20%	63.68%
	RA	0.33%	65.09%
	LP	0.96%	71.46%
	LB	0.59%	70.93%
average	overall	0.52%	67.79%

**Table 6:** BD performance and time reduction comparisons of three methods encoded with AI configuration.

Anchor(VTM5.0) SAO Disabled with 128 × 128 CTU		Proposed		Sobel-Based EO [27]		Depth-Based EO [28]		Spatial Correlation-Based [30]	
		Y BD-Rate	ΔT	Y BD-Rate	ΔT	Y BD-Rate	ΔT	Y BD-Rate	ΔT
Sequence Class	ClassB	0.15%	63.22%	0.12%	50.76%	0.07%	10.40%	0.12%	38.41%
	ClassC	0.03%	62.87%	0.02%	53.43%	0.05%	2.90%	0.06%	51.47%
	ClassD	−0.04%	63.01%	0.01%	56.65%	0.35%	3.10%	0.04%	45.91%
	ClassE	0.10%	64.43%	0.11%	52.42%	0.12%	14.80%	0.15%	50.55%
	ClassF	0.77%	64.87%	0.42%	53.89%	0.73%	16.90%	0.36%	55.34%
average	overall	0.20%	63.68%	0.14%	53.43%	0.26%	9.62%	0.15%	48.34%

## References

[1] Cordeiro P.J., Assunçãa P. (2012). Distributed Coding/Decoding Complexity in Video Sensor Networks. Sensors.

[2] Pan Z., Chen L., Sun X. (2015). Low Complexity HEVC Encoder for Visual Sensor Networks. Sensors.

[3] Wu D., Shi H., Wang H., Wang R., Fang H. (2019). A Feature-Based Learning System for Internet of Things Applications. IEEE Internet Things J..

[4] Meneghello F., Calore M., Zucchetto D., Polese M., Zanella A. (2019). IoT: Internet of Threats? A Survey of Practical Security Vulnerabilities in Real IoT Devices. IEEE Internet Things J..

[5] Zarca A.M., Bernabe J.B., Skarmeta A., Calero J.M.A. (2020). Virtual IoT HoneyNets to Mitigate Cyberattacks in SDN/NFV-Enabled IoT Networks. IEEE J. Sel. Areas Commun..

[6] Hafeez I., Antikainen M., Ding A.Y., Tarkoma S. (2020). IoT-KEEPER: Detecting Malicious IoT Network Activity Using Online Traffic Analysis at the Edge. IEEE Trans. Netw. Serv. Manag..

[7] Wu D., Zhang Z., Wu S., Yang J., Wang R. (2019). Biologically Inspired Resource Allocation for Network Slices in 5G-Enabled Internet of Things. IEEE Internet Things J..

[8] Nightingale J., Salva-Garcia P., Calero J.M.A., Wang Q. (2018). 5G-QoE: QoE Modelling for Ultra-HD Video Streaming in 5G Networks. IEEE Trans. Broadcast..

[9] Tang T., Yang J., Du B., Tang L. (2019). Down-Sampling Based Rate Control for Mobile Screen Video Coding. IEEE Access.

[10] Tang T., Du B., Tang L., Yang J., He P. (2019). Distortion Propagation Based Quantization Parameter Cascading Method for Screen Content Video Coding. IEEE Access.

[11] Sullivan G.J., Ohm J., Han W., Wiegand T. (2012). Overview of the High Efficiency Video Coding (HEVC) Standard. IEEE Trans. Circuits Syst. Video Technol..

[12] Filippov A., Rufitskiy V., Chen J., Alshina E. Intra Prediction in the Emerging VVC Video Coding Standard. Proceedings of the 2020 Data Compression Conference (DCC).

[13] Amestoy T., Mercat A., Hamidouche W., Menard D., Bergeron C. (2020). Tunable VVC Frame Partitioning Based on Lightweight Machine Learning. IEEE Trans. Image Process..

[14] Perfecto C., Elbamby M.S., Ser J.D., Bennis M. (2020). Taming the Latency in Multi-User VR 360°: A QoE-Aware Deep Learning-Aided Multicast Framework. IEEE Trans. Commun..

[15] Costa M., Moreira R., Cabral J., Dias J., Pinto S. (2020). Wall Screen: An Ultra-High Definition Video-Card for the Internet of Things. IEEE MultiMedia.

[16] Fan Y., Chen J., Sun H., Katto J., Jing M. (2020). A Fast QTMT Partition Decision Strategy for VVC Intra Prediction. IEEE Access.

[17] Fu C., Alshina E., Alshin A., Huang Y., Chen C., Tsai C., Hsu C., Lei S., Park J., Han W. (2012). Sample Adaptive Offset in the HEVC Standard. IEEE Trans. Circuits Syst. Video Technol..

[18] Alshin A., Alshina E., Park J. Sample Adaptive Offset Design in HEVC. Proceedings of the 2013 Data Compression Conference.

[19] Baldev S., Shukla K., Gogoi S., Rathore P.K., Peesapati R. (2018). Design and Implementation of Efficient Streaming Deblocking and SAO Filter for HEVC Decoder. IEEE Trans. Consum. Electron..

[20] Choi Y., Joo J. (2015). Exploration of Practical HEVC/H.265 Sample Adaptive Offset Encoding Policies. IEEE Signal Process. Lett..

[21] Saldanha M., Sanchez G., Marcon C., Agostini L. Complexity Analysis Of VVC Intra Coding. Proceedings of the 2020 IEEE International Conference on Image Processing (ICIP).

[22] Pakdaman F., Adelimanesh M.A., Gabbouj M., Hashemi M.R. Complexity Analysis Of Next-Generation VVC Encoding And Decoding. Proceedings of the 2020 IEEE International Conference on Image Processing (ICIP).

[23] Aklouf M., Leny M., Dufaux F., Kieffer M. Low Complexity Versatile Video Coding (VVC) for Low Bitrate Applications. Proceedings of the 2019 8th European Workshop on Visual Information Processing (EUVIP).

[24] Usman M.A., Usman M.R., Shin S.Y. (2018). Exploiting the Spatio-Temporal Attributes of HD Videos: A Bandwidth Efficient Approach. IEEE Trans. Circuits Syst. Video Technol..

[25] Pan X. (2020). Fusing HOG and convolutional neural network spatial-temporal features for video-based facial expression recognition. IET Image Process..

[26] Joo J., Choi Y., Lee K. Fast sample adaptive offset encoding algorithm for HEVC based on intra prediction mode. Proceedings of the 2013 IEEE Third International Conference on Consumer Electronics Berlin (ICCE-Berlin).

[27] Joo J., Choi Y. Dominant edge direction based fast parameter estimation algorithm for sample adaptive offset in HEVC. Proceedings of the 2014 IEEE International Conference on Image Processing (ICIP).

[28] Zhengyong Z., Zhiyun C., Peng P. A fast SAO algorithm based on coding unit partition for HEVC. Proceedings of the 2015 6th IEEE International Conference on Software Engineering and Service Science (ICSESS).

[29] Gendy S.E., Shalaby A., Sayed M.S. Fast parameter estimation algorithm for sample adaptive offset in HEVC encoder. Proceedings of the 2015 Visual Communications and Image Processing (VCIP).

[30] Kim S., Jeong J., Moon J., Kim Y. Fast sample adaptive offset parameter estimation algorithm based on early termination for HEVC encoder. Proceedings of the 2017 IEEE International Conference on Consumer Electronics (ICCE).

[31] Zhang W., Guo C. Design and implementation of parallel algorithms for sample adaptive offset in HEVC based on GPU. Proceedings of the 2016 Sixth International Conference on Information Science and Technology (ICIST).

[32] de Souza D.F., Ilic A., Roma N., Sousa L. HEVC in-loop filters GPU parallelization in embedded systems. Proceedings of the 2015 International Conference on Embedded Computer Systems: Architectures, Modeling, and Simulation (SAMOS).

[33] Wang Y., Guo X., Lu Y., Fan X., Zhao D. GPU-based optimization for sample adaptive offset in HEVC. Proceedings of the 2016 IEEE International Conference on Image Processing (ICIP).

[34] Wang Y., Guo X., Fan X., Lu Y., Zhao D., Gao W. (2018). Parallel In-Loop Filtering in HEVC Encoder on GPU. IEEE Trans. Consum. Electron..

[35] Zhang Q., Wang Y., Huang L., Jiang B. (2020). Fast CU Partition and Intra Mode Decision Method for H.266/VVC. IEEE Access..

[36] Huang Y., Hsu C., Chen C., Chuang T., Hsiang S., Chen C., Chiang M., Lai C., Tsai C., Su Y. (2020). A VVC Proposal With Quaternary Tree Plus Binary-Ternary Tree Coding Block Structure and Advanced Coding Techniques. IEEE Trans. Circuits Syst. Video Technol..

[37] Zhang K., Chen J., Zhang L., Li X., Karczewicz M. (2018). Enhanced Cross-Component Linear Model for Chroma Intra-Prediction in Video Coding. IEEE Trans. Image Process..

[38] Abdoli M., Henry F., Brault P., Duhamel P., Dufaux F. (2018). Short-Distance Intra Prediction of Screen Content in Versatile Video Coding (VVC). IEEE Signal Process. Lett..

[39] Zhang K., Zhang L., Chien W., Karczewicz M. (2019). Intra-Prediction Mode Propagation for Video Coding. IEEE J. Emerg. Sel. Top. Circuits Syst..

[40] Bossen F., Boyce J., Li X., Seregin V., Shring K. JVET common test conditions and software reference configurations for SDR video. Proceedings of the Document JVET-N1010, 14th JVET Meeting.

[41] Rosewarne C., Sharman K., Flynn D. (2014). Common Test Conditions and Software Reference Configurations for HEVC Range Extensions.

[42] Bjϕntegaard G. Document VCEG-M33: Calculation of Average PSNR Differences between RD-Curves. Proceedings of the ITU-T VCEG Meeting.

